# Establishing a Split Luciferase Assay for Proteinkinase G (PKG) Interaction Studies

**DOI:** 10.3390/ijms19041180

**Published:** 2018-04-12

**Authors:** Andrea Schramm, Philip Mueller-Thuemen, Timo Littmann, Manuela Harloff, Takeaki Ozawa, Jens Schlossmann

**Affiliations:** 1 Department of Pharmacology and Toxicology, University of Regensburg, 93053 Regensburg, Germany; Andrea.Schramm@ur.de (A.S.); philip.mueller-thuemen@bsse.ethz.ch (P.M.-T.); Manuela.Harloff@ur.de (M.H.); 2 Department of Biosystems Science and Engineering, ETH Zurich, 4058 Basel, Switzerland; 3 Department of Pharmaceutical and Medicinal Chemistry II, University of Regensburg, 93053 Regensburg, Germany; Timo.Littmann@ur.de; 4 Department of chemistry, School of Science, University of Tokyo, Tokyo 113-0033, Japan; ozawa@chem.s.u-tokyo.ac.jp

**Keywords:** PKG, cGK, RGS2, cGMP, PCA, luciferase, protein–protein interaction

## Abstract

Nitric oxide (NO/cyclic guanosine monophosphate (cGMP)-regulated cellular mechanisms are involved in a variety of (patho-) physiological processes. One of the main effector molecules in this system, proteinkinase G (PKG), serves as a molecular switch by phosphorylating different target proteins and thereby turning them on or off. To date, only a few interaction partners of PKG have been described although the identification of protein–protein interactions (PPI) is indispensable for the understanding of cellular processes and diseases. Conventionally used methods to detect PPIs exhibit several disadvantages, e.g., co-immunoprecipitations, which depend on suitable high-affinity antibodies. Therefore, we established a cell-based protein-fragment complementation assay (PCA) for the identification of PKG target proteins. Here, a reporter protein (*click beetle* luciferase) is split into two fragments and fused to two different possible interaction partners. If interaction occurs, the reporter protein is functionally complemented and the catalyzed reaction can then be quantitatively measured. By using this technique, we confirmed the regulator of G-Protein signaling 2 (RGS2) as an interaction partner of PKGIα (a PKG-isoform) following stimulation with 8-Br-cGMP and 8-pCPT-cGMP. Hence, our results support the conclusion that the established approach could serve as a novel tool for the rapid, easy and cost-efficient detection of novel PKG target proteins.

## 1. Introduction

Protein–protein interactions (PPI) regulate a huge variety of cellular processes, e.g., DNA replication, diverse transport mechanisms and signal transduction networks. The entirety of all PPIs (referred to as the interactome) of an organism was estimated in 2008, and approximately 650k interactions were calculated [1]. The identification of the structure and regulation of PPIs is an indispensable necessity, not only for the understanding of basic principles in cellular processes but also for the elucidation of various diseases, as these are often related to altered PPIs. Methods to detect PPIs range from classical in vitro procedures such as co-immunoprecipitation to modern in vivo assays like the yeast two-hybrid system (Y2H), FRET and BRET (Foerster/bioluminescence resonance energy transfer) as well as molecular display methods (e.g., phage displays). However, these approaches all show certain disadvantages; for instance, the application of Y2H is restricted to proteins, which are transportable into the nucleus. Therefore, PPIs of, e.g., membrane proteins, cannot be detected with Y2H. The use of protein-fragment complementation assays (PCA) can help to circumvent this problem, as it enables the discovery of PPIs in all relevant subcellular compartments/organelles. For this purpose, two per se inactive fragments of a reporter protein are fused with two possible interaction partners. If the target proteins interact with each other, both reporter fragments are arranged in close proximity and are spontaneously reconstituted to form a functional protein. The decision which reporter protein to choose should be made with respect to the following considerations: At first, the protein should have a small molecular weight and be monomeric. Moreover, it should not be expressed in the used cell line (or at least there has to be a possibility to inhibit endogenous expression). If overexpressed, the protein of choice must not be toxic for cells. And last, simple in vitro/in vivo detection methods have to be available. One of the first described PCA strategies involved ubiquitin-based split protein sensors [2,3]. Other non-enzymatic PCA systems are based on the use of fluorescent proteins like green fluorescent protein (GFP). Nonetheless, as the complementation of these fluorescent reporters is irreversible, it is not possible to analyze the kinetics of the investigated PPI [4]. In contrast, enzyme-based PCA not only allows the dissociation of the reporter protein fragments and hence, a real-time analysis of the analyzed interactions, but also a marked signal amplification. Accordingly, the fusion proteins do not have to be overexpressed and the risk of self-association of both fragments is significantly lowered compared to fluorescent fragments [5]. Used reporter enzymes include, for example, dihydrofolate reductase, β-galactosidase and β-lactamase [6,7,8]. Nowadays, luciferases are among the most popular reporter proteins, although the signal depends on adding a specific substrate. Nonetheless, the specific properties of luciferases allow a versatile application in PCAs: first, luciferases are composed of independent subdomains so that association and dissociation becomes reversible [9]. Next, the quick folding of the fragments as well as the characteristic signal amplification makes them an ideal candidate to prove weak and transient PPIs [10]. Last, luciferase-based PCA systems enable detection, characterization and localization of regulated or induced PPIs in cells and living organisms in real-time [11,12]. Luciferases from different organisms show different properties. *Renilla*-luciferases need coelenterazine as a substrate, which is quite unstable in air, and emits blue light (λ_max_: 475 nm) spectral conditions, which are rather unfavorable [13]. In contrast, *firefly* and *click beetle* luciferases use d-luciferin, a more stable substrate, and emit a more long-wave light (yellow to red, λ_max_: 575–600 nm). Moreover, *click beetle* luciferases emit 10 times more photons compared to *firefly* luciferases, so that the signal becomes brighter [13]. Finally, the emission spectrum of *click beetle* luciferases is pH-independent, making it the ideal candidate for the detection of PPIs in vivo [14].

Here, we established a *click beetle* luciferase complementation assay to investigate interactions between proteinkinase G, isoform Iα (PKGIα) and possible interaction partners using the example of regulator of G-protein signaling 2 (RGS2). PKGs, also referred to cGMP dependent kinases (cGKs), are coded by two different genes (*prkg1* and *prkg2*). The *prkg1*-mRNA is alternatively spliced, so that this gene gives rise to two different cytosolic isoforms of cGKI (α- and β-isoform, respectively). In contrast, *prkg2* codes for PKGII/cGKII, which is located at the intracellular site of the plasma membrane by myristoylation [15]. Considering cGKI-isoforms, these proteins mainly differ in the first 100 amino acids of the N-terminal region and exhibit a rod-like structure which is composed of a regulatory and catalytic subunit [16,17,18]. All cGKs show a specific expression pattern and are responsible for a diversity of cellular functions including vasorelaxation (for detailed information about expression and function of all isoforms please refer to [19]). Before acting on different substrates, cGKs, which already exist as a homodimer in the inactive state, must be activated by the second messenger cGMP, which mediates possibly stronger interactions of the dimer [20,21]. More important, cGMP induces a conformational change in the protein, so that it is no longer auto inhibited [20]. Following activation, the kinase is able to bind specific substrates with its catalytical domain and transfer the terminal phosphate from the donor ATP to serine or threonine residues in the substrate. Other publications point to an alternative way to activate cGKIα: oxidants can induce an intermonomeric disulfide-bond at cysteine 42 so that the intermolecular interaction of both monomers of the kinase is enhanced and the dimer becomes more stable. By forming this disulfide-bond, cGKIα might be activated without the involvement of cGMP, a suggestion that has been controversially discussed in the past few years [22,23,24,25].

In 2003, Tang et al. identified RGS2 as a binding partner and phosphorylation target of cGKIα [26]. These negative regulators of G-protein-coupled receptors (GPCR) accelerate hydrolysis of G-protein-bound GTP and, therefore, terminate GPCR-induced signals [27]. Performing extensive studies, Tang et al. showed that cGKIα binds and phosphorylates RGS2 at its N-terminal region. Afterwards, the phosphorylated RGS2 translocates to the cell membrane to interact with Gα_q/11_-subunits, which leads in turn to the termination of IP_3_-mediated [Ca^2+^]_i_ increase and, hence, to vasodilation [26]. Actually, RGS2-knockout mice display a hypertensive phenotype and prolonged vasoconstrictor signaling, impressively demonstrating the important role of RGS2 in blood pressure regulation [28]. However, the studies of Tang et al. provide only in vitro evidence for the cGKIα/RGS2 interaction, as they were using either purified cGKIα or recombinant RGS2. Some years later, it was demonstrated in vivo that the phosphorylation of RGS2 by cGKIα is indispensable for the association of RGS2 with the plasma membrane [29]. Nonetheless, these authors did not confirm the direct interaction of cGKIα and RGS2 in vivo. By establishing a cell-based luciferase complementation assay, we now further substantiate these previous findings regarding a direct interaction of cGKIα and RGS2 in vivo. Moreover, this assay is a valuable tool in future studies regarding the identification of new PKG-substrates and extends existing methods like co-immunoprecipitation or cGMP-agarose affinity purification, which are dependent on stable interactions between the two proteins.

## 2. Results

### 2.1. Construction of Vectors

**Figure 1:**
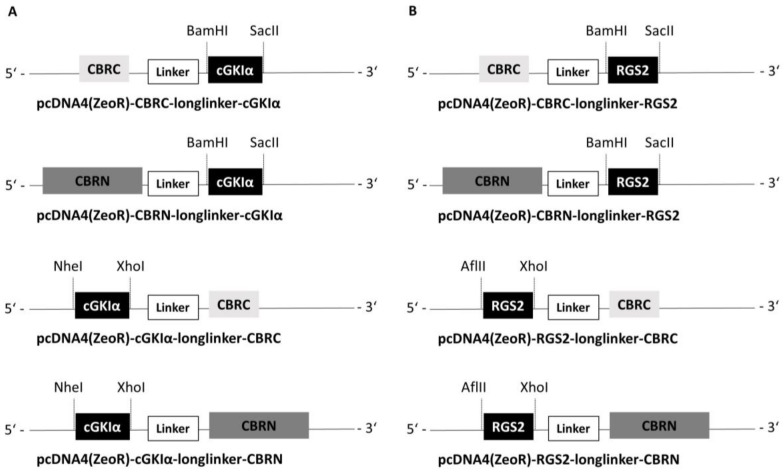
Schematic illustration of cloned vectors. Used restriction enzymes and restriction sites are indicated with dashed lines. (**A**) cGKIα vectors (**B**) RGS2 vectors; linker consists of 17 Gly and Ser residues.

As a basis for analyzing protein–protein interactions, we took advantage of the investigations of Villalobos et al. and Hida et al., who showed that luciferase from *click beetle* (emitting red light = CBR), with respect to reassembling and complementation, can be ideally split up in a large N-terminal fragment consisting of aa 1–416 (CBRN) and a small C-terminal fragment consisting of aa 395–542 (CBRC), whereby the sequence overlap is necessary for reconstitution of both fragments [30,31,32]. We therefore cloned the cDNA of cGKIα and subsequently of RGS2 into pcDNA-vectors, which already contained the cDNA for either CBRN or CBRC (full-length CBR vectors were kindly provided by T. Ozawa, CBRN and CBRC split vectors were generated by Timo Littmann; primers with used restriction sites can be found in Table S1). The attached protein can be located either at the N-terminus or at the C-terminus of both CBRC and CBRN, and accordingly, we constructed 4 vectors for each protein (cGKIα and RGS2, respectively). The luciferase fragments were separated from cGKIα and RGS2, respectively, by flexible linkers consisting of Gly and Ser residues with a total length of 17 amino acids. A schematic illustration of all vectors including used restriction sites can be found in Figure 1. For better clarity, vectors are abbreviated hereafter as CBRC-cGKIα, cGKIα-CBRC, CBRN-cGKIα and cGKIα-CBRN, likewise for RGS2-vectors, foregoing linker, resistance to antibiotics etc. Finally, vectors were sequenced to verify correct sequences.

### 2.2. Expression in a Eukaryotic System

**Figure 2:**
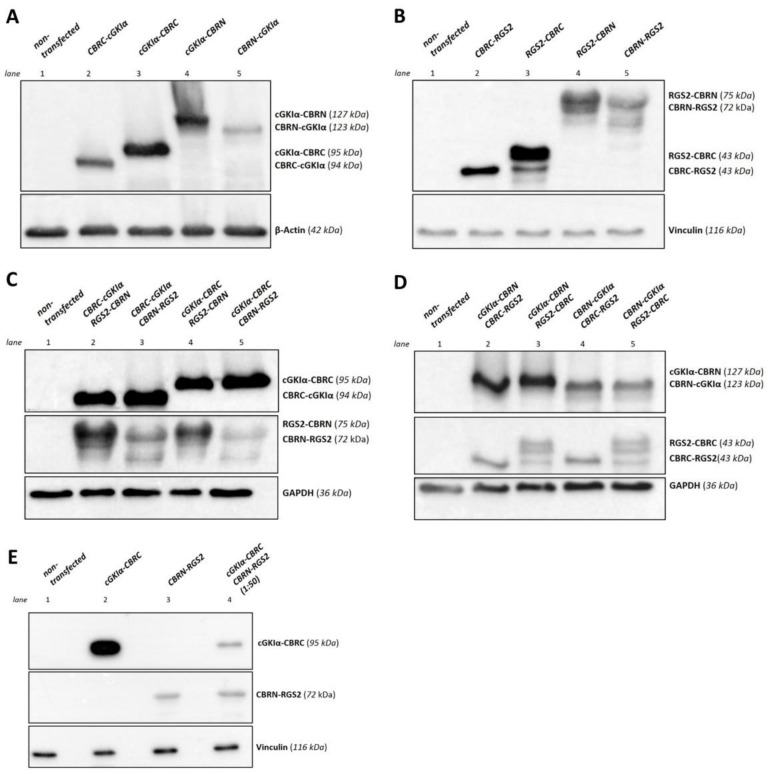
Expression control of cGKIα- and RGS2- fusion proteins. COS-7-cells were transfected with the appropriate vector (22.5 µg overall DNA amount in 75 cm^2^ cell culture flasks). After cell lysis, 50 µg of lysate were loaded on each lane and a Western blot was performed. Fusion proteins of cGKIα and RGS2 with CBR-fragments were detected using cGKIα- or RGS2-antibodies. (**A**) Expression of cGKIα-fusion proteins. Proteins were separated using an 11.5% sodium dodecyl sulfate polyacrylamide gel electrophoresis (SDS-PAGE). β-Actin served as loading control. All possible fusion proteins were found to be expressed; (**B**) Expression of RGS2-fusion proteins. Proteins were separated using a 12.5% SDS-PAGE. Vinculin served as loading control. All possible fusion proteins were found to be expressed; (**C**,**D**) Co-expression of cGKIα- and RGS2-fusion proteins. Each vector pair was co-transfected using a 1:1 vector ratio. Proteins were separated using a 11.5% SDS-PAGE. Glyceraldehyde 3-phosphate dehydrogenase (GAPDH) served as loading control. All possible fusion proteins were found to be expressed, while expression of cGKIα-vectors was much more intense in all tested conditions, so that exposure time for RGS2-fusion proteins needed to be adjusted; (**E**) Co-expression of cGKIα- and RGS2-fusion proteins with adjusted vector ratio. The vector pair cGKIα-CBRC and CBRN-RGS2 was co-transfected using a 1:50 vector ratio. Proteins were separated using an 11.5% SDS-PAGE. Vinculin served as loading control. In contrast to (**C**,**D**), exposure time for cGKIα- and RGS2-fragments did not differ indicating that proteins were expressed in an equimolar range. kDa: calculated molecular weight of respective fusion proteins.

Next, we checked, if the expected fusion proteins can be expressed in a eukaryotic system. Thus, we transfected COS-7-cells either with 22.5 µg of one single vector or with different combinations of cGKIα- and RGS2-vectors with 11.25 µg of each vector (vector ratio 1:1) by means of calcium-phosphate transfection. As control, we used non-transfected cells. After cell lysis, we performed Western blots to verify expression of both cGKIα- and RGS2-CBR-fusion proteins, whereas detection was performed with selective antibodies against cGKIα or RGS2. As shown in Figure 2, all cGKIα- (Figure 2A) or RGS2-fusion proteins (Figure 2B) were expressed following transfection of COS-7-cells. As expected, if proteins were fused to the CBRC-fragment, they appeared at a smaller molecular weight compared to CBRN-fused proteins (expected molecular weights as calculated are assigned on the right side of each blot). Although proteins, which were fused either with N-terminal or C-terminal to CBRC or CRBN should have appeared at a similar molecular weight; in fact, they differed slightly relating to their gel-running behavior. Apparently, protein folding of these fusion proteins was variable depending on where the luciferase-tag was located. Afterwards, we analyzed all possible combinations of cGKIα- and RGS2-vectors (Figure 2C,D). Interestingly, all combinations of fusion proteins were expressed. As cGKIα-fusion proteins with the small CBR-fragment (CBRC) appeared to be expressed more strongly than CBRN-fused proteins (Figure 2C), we focused on combinations only with RGS2-fusion proteins containing the larger fragment (CBRN) in the following interaction experiments. Interestingly, some RGS2-fragments could not be detected as single bands (e.g., RGS2-CBRC, Figure 2D). RGS2-mRNA contains four different translation initiation sites and gives rise to a set of different isoforms; therefore, the observed bands could be due to alternative splicing of the RGS2-fragments [33]. However, expression of cGKIα-fusion proteins was much stronger in all tested combinations, so that a prolonged exposure time was needed to visualize RGS2-fusion proteins also. An equimolar amount of both interaction partners is an important prerequisite for analyzing a possible interaction of cGKIα and RGS2 via PCA. Given this, we needed to adjust the vector ratio in the following experiments.

### 2.3. Interaction Analysis of cGKIα and RGS2

Before we could analyze the interaction between cGKIα and RGS2, we needed to establish different controls for our experiments. As negative controls, we used four different settings, where we either transfected both vectors without transfection reagent, transfected only one vector, or there was no DNA at all (water control). As expected, we could not reveal any signal amplification following stimulation with 8-Br-cGMP under these conditions (shown for one vector combination in Figure S1A). To prove if the system was actually working, we made use of the well-known rapamycin-mediated interaction between FKBP and FRB (FK506 binding protein and FKBP-and rapamycin binding domain of mTOR) [30] (Figure S1B). We detected a 14-fold signal amplification 24 h after rapamycin induction. Signal amplification was even more pronounced (38-fold) 48 h after stimulation, possibly due to a lower basal signal and more time for protein expression. Hence, FKBP/FRB-interaction was a suitable positive control, so that positive and negative controls were carried out in all consecutive experiments, however, non-explicitly shown in the herein presented figures.

**Figure 3:**
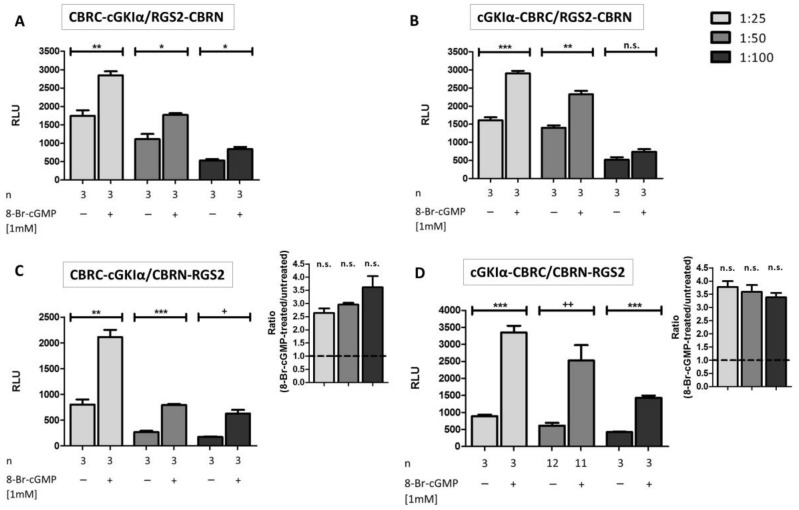
Influence of vector ratio for transfection and subsequent interaction analysis of cGKIα and RGS2: COS7-cells were seeded in 6-well plates (3.3 × 10^5^ cells/well) and co-transfected with 4 different combinations of cGKIα/RGS2-vectors (each transfection with 15 µg DNA, vector ratio as indicated). After transfer to 96-well plates (1.0 × 10^4^ cells/well) and addition of 1 mM 8-Br-cGMP, cells were incubated for 24 h. In most cases, a significant signal-increase was observed (**A**–**D**). The most promising vector combinations for enhanced luminescence upon 8-Br-cGMP application were CBRC-cGKIα/CBRN-RGS2 (**C**) and cGKIα-CBRC/CBRN-RGS2 (**D**); ratiometric analysis (8-Br-cGMP-treated/untreated) can be seen on the inset of (**C**,**D**), respectively. Data is expressed as mean ± standard error of the mean (SEM). For unpaired Student’s *t*-test *p*-values < 0.05 were considered significant (*, ^+^), <0.01 and <0.001 highly significant (**, ^++^ and ***, respectively), whereas statistical differences characterized by an ^+^ were calculated by unpaired Student’s *t*-test with Welch’s correction. A non-significant difference was marked as n.s. N = technical replicates. RLU: relative luminescence unit.

To determine the optimal vector ratio for cGKIα:RGS2 vectors, we examined different vector ratios ranging from 1:2 to 1:10. However, we were not able to detect any signal amplifications following 8-Br-cGMP stimulation [34]. We concluded that expression of RGS2-fusion proteins was still too low under these conditions, so we further intensified vector ratios to 1:25, 1:50 and 1:100; moreover, we also tried all combinations of the different vectors of Figure 1 to find out the optimal vector combination (Figure 3A–D). We revealed a significant signal amplification in almost all tested cases compared to basal luminescence levels 24 h post stimulation. However, vector ratios of 1:25 to 1:50 produced more pronounced increases compared to 1:100 vector ratios. Apparently, the position of the CBRC fragment fused to cGKIα was not an issue, as luminescence signals were quite similar between N-terminal and C-terminal fused fragments (Figure 3A,C vs. Figure 3B,D). In contrast, at least basal values of unstimulated cells were notably smaller if the CBRN-fragment was fused in an N-terminal position to RGS2 (Figure 3C,D). Possibly, the C-terminal tagged RGS2 (RGS2-CBRN) has a different binding capacity towards cGKIα and is therefore non-specifically interacting. The luminescence signal did not differ much after cGMP stimulation in all tested conditions. Altogether, the combination cGKIα-CBRC/CBRN-RGS2 led to the highest amplifications (ratiometric analysis on the inset of C and D, approximately 3.5-fold amplification compared to basal luminescence). In contrast to the positive control, a longer exposure time of the stimulus (48 h) had no influence on signal amplification (Figure S2). Presumably, the cGMP-cGKIα-RGS2 system is already in steady-state conditions after 24 h. In preliminary experiments, the vector combination cGKIα-CBRC/CBRN-RGS2 in a vector ratio of 1:50 produced the highest signal amplification (not shown). However, with ongoing experiments it turned out, that a 1:25 ratio led to an even higher luminescence signal, albeit the basal luminescence signal without stimulation with cGMP was also slightly enhanced. We checked the signal ratio of cGMP treated to untreated cells of both conditions (1:25 and 1:50 transfected cells) which did not differ significantly (Figure 3C,D, inset). When analyzing the protein-expression levels, we found that a transfection ratio of 1:50 led to an almost equimolar expression of cGKIα-CBRC and CBRN-RGS2 (Figure 2E). As this vector combination produced the lowest background signal and led to a 3.5-fold signal amplification after cGMP-stimulation, we conducted all further experiments with these vectors in a transfection ratio of 1:50 (cGKIα-CBRC:CBRN-RGS2), with a stimulus incubation time of 24 h.

### 2.4. Comparison of Expression Level of cGKIα with Tissue Expression and Analysis of Activity

**Figure 4:**
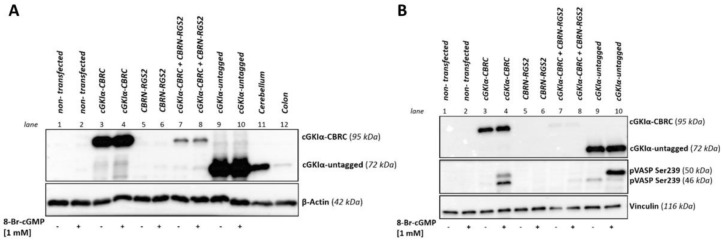
Comparison of expression level and activity of CBRC-tagged cGKIα. COS7-cells were seeded in 75 cm^2^ culture flasks and either transfected with one vector or co-transfected with the vector pair cGKIa-CBRC/CBRN-RGS2 (22.5 µg DNA, 1:50). 48 h post transfection, cells were either stimulated with 1 mM 8-Br-cGMP for 1 h or left untreated. After cell lysis, 50 µg of lysate were loaded on each lane and an 11.5% SDS-PAGE followed by Western blot was performed. cGKIα (tagged with CBRC or untagged) was detected using cGKIα-antibodies. (**A**) Comparison of expression level: murine wild-type cerebellum and colon served as positive tissue control (50 µg each). In contrast to single transfections of either cGKIα-CBRC or untagged cGKIα, the expression level of cGKIα-CBRC when co-transfected with CBRN-RGS2 is not enhanced compared to tissue controls. β-actin served as loading control; (**B**) Analysis of activity: activity of cGKIα was shown using a p-Ser239-VASP antibody, vinculin served as loading control. Stimulation with cGMP caused a strong p-Ser239 VASP signal when cells were transfected with cGKIα-CBRC or untagged cGKIα alone. When cells were co-transfected with cGKIα-CBRC/CBRN-RGS2, a weak p-Ser239 VASP signal could be detected. VASP: vasodilator-stimulated phosphoprotein.

To check whether the expression levels of cGKIα-CBRC strongly exceed tissue concentrations of the native protein and, therefore, could account for a false-positive signal, we transfected cells either with cGKIα-CBRC alone, with cGKIα-CBRC and CBRN-RGS2 in combination, or with a vector containing an untagged and, therefore, native sequence of cGKIα. We performed a Western blot and compared the protein expression of transfected cells to the protein expression of native cGKIα in murine tissue lysate either known to strongly express cGKIα (cerebellum) or where only a weak expression of native cGKIα can be detected (colon), Figure 4A. While transfection with cGKIα-CBRC alone as well as with untagged cGKIα leads to a strong overexpression compared to tissue lysates, the co-transfected cells expressed cGKIα-CBRC at a level somewhere in between the weak and the strong tissue expression. Hence, the established assay reflects protein expression also found in physiological conditions. Moreover, we were also interested to see if the CBRC-tag affects activity of the kinase (Figure 4B). Therefore, we either stimulated pre-transfected cells with 1 mM 8-Br-cGMP or left them untreated. pVASP-Ser 239 was used to reveal cGKIα-activity. VASP (vasodilator-stimulated phosphoprotein) is a known substrate of different kinases, which is expressed among others in platelets and smooth muscle cells. Phosphorylation can occur at two different phosphorylation sites (Ser 157; Ser 239) [35,36]. The 46 kDa- band of the Ser 239 phosphorylated protein shifts in a sodium dodecyl sulfate polyacrylamide gel electrophoresis (SDS-PAGE) to a 50 kDa band upon Ser 157 phosphorylation. We detected a strong VASP phosphorylation at Ser 239 upon cGMP-stimulation in cells transfected with either untagged (control-) cGKIα or with cGKIα-CBRC alone (lane 3/4 and lane 9/10). When both interaction partners where transfected (cGKIα-CBRC + CBRN-RGS2, lane 7/8), we still observed a faint band, yet the signal was much weaker compared to single transfections. We assumed that the decrease in phosphorylation intensity was due to the strongly reduced cGKIα-CBRC protein expression (since only 1/50 of DNA amount of cGKIα-CBRC was used in the co-transfections compared to single transfections). However, the CBRC-tag does not interfere with activity of the kinase, as we detected a massive VASP phosphorylation at Ser 239 upon cGMP stimulation, at least in cells single-transfected with cGKIα-CBRC.

### 2.5. Comparison of Different Stimuli

**Figure 5:**
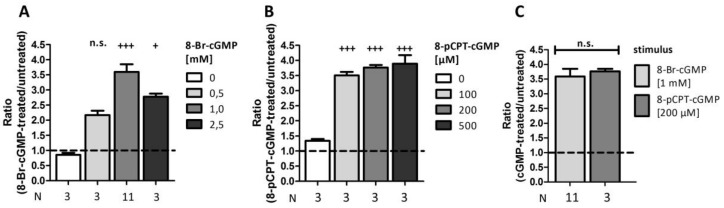
Comparison of different stimuli. COS7-cells were seeded in 6-well plates (3.3 × 10^5^ cells/well) and co-transfected with the vector pair cGKIα-CBRC/CBRN-RGS2 (15 µg DNA, 1:50). After transfer on 96-well plates (1.0 × 10^4^ cells/well) cells were stimulated with different concentrations of either 8-Br-cGMP (**A**) or 8-pCPT-cGMP (**B**) and incubated for 24 h. (**C**) compares the ratios obtained upon incubation with 8-Br-cGMP (1 mM) and 8-pCPT-cGMP (200 µM). Data is expressed as mean ± SEM. For *one-Way*-ANOVA *p*-values < 0.05 were considered significant (^+^) < 0.001 highly significant (^+++^). A non-significant difference was marked as n.s. N = technical replicates. Dashed line: signal-ratio = 1.

Besides 8-Br-cGMP, there is also another cGMP-analog (8-pCPT-cGMP) available. Thus, we investigated whether these two analogs in varying concentrations caused differences in signal amplification (Figure 5). Ratiometric analysis revealed a highly significant 3.6 ± 0.3-fold signal amplification following incubation with 1 mM 8-Br-cGMP, whereas higher or lower concentrations were not as efficient (0.5 mM: 2.2 ± 0.2-fold amplification, 2.5 mM: 2.8 ± 0.1-fold amplification; Figure 5A). Interestingly, already a 10 times lower concentration of 8-pCPT-cGMP led to the same increase in luminescence compared to stimulation with 1 mM 8-Br-cGMP; we could not detect a considerable increase by using higher concentrations (100 µM: 3.5 ± 0.1-fold amplification, 200 µM: 3.8 ± 0.1-fold amplification, 500 µM: 3.9 ± 0.3-fold amplification; Figure 5B). As 1 mM 8-Br-cGMP and 200 µM 8-pCPT-cGMP are often used concentrations in literature, we directly compared the signal ratios via an unpaired Student’s *t*-test (Figure 5C). However, we did not discover a significant difference, indicating that both cGMP-analogs can be used in this PCA in the given concentrations.

### 2.6. Analysis of Specificity

**Figure 6:**
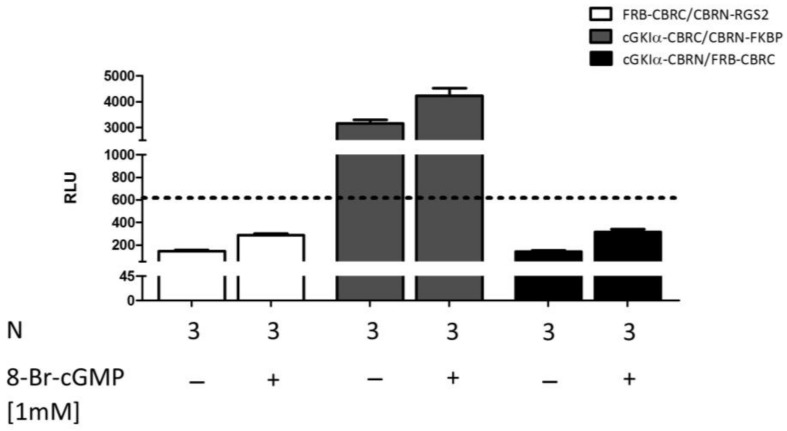
Analysis of specificity. COS7-cells were seeded in 6-well plates (3.3 × 10^5^ cells/well) and co-transfected with different negative controls (in all cases 15 µg of DNA in a vector ratio of 1:50). After transfer to 96-well plates (1.0 × 10^4^ cells/well) cells were stimulated with 8-Br-cGMP and incubated for 24 h. As a reference, mean value of unstimulated cells which were transfected with cGKIα-CBRC/CBRN-RGS2 was used (dashed line, RLU = 618). Data is expressed as mean ± SEM. N = technical replicates. RLU: relative luminescence unit.

To exclude the possibility of a spontaneous complementation of the luciferase fragments and, therefore, to prove the specificity of the established PCA systems, we added further negative controls. Next to the already mentioned controls in Figure S1A, we also examined vector combinations of proteins, which are so far not known to interact with each other (RGS2 with FRB and cGKIα with FRB or FKBP) (Figure 6). Co-transfection of FRB-CRBC and CBRN-RGS2 as well as cGKIα-CBRN and FRB-CBRC did not reveal any signals above reference (which consisted of mean value of unstimulated cells co-transfected with the cGKIα-CBRC/CBRN-RGS2 vector combination). In contrast, when we co-transfected cells with cGKIα-CBRC and CBRN-FKBP and left them untreated, we observed a luminescence intensity almost comparable to stimulated cells co-transfected with cGKIα-CBRC/CBRN-RGS2 (3158 ± 137 relative luminescence units (RLU)). The signal was further enhanced by stimulation with 8-Br-cGMP (4231 ± 293 RLU). Accordingly, we concluded that both proteins might interact non-specifically with each other.

### 2.7. Analysis of Selectivity

**Figure 7:**
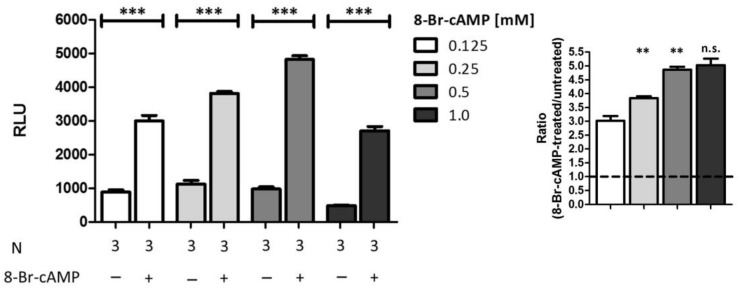
Analysis of selectivity. COS7-cells were seeded in 6-well plates (3.3 × 10^5^ cells/well) and co-transfected with the vector pair cGKIα-CBRC/CBRN-RGS2 (15 µg DNA, 1:50). After transfer on 96-well plates (1.0 × 10^4^ cells/well) cells were stimulated with different concentrations of 8-Br-cAMP and incubated for 24 h. Data is expressed as mean ± SEM. For unpaired Student’s *t*-test *p*-values < 0.01 and < 0.001 were considered highly significant (** and ***, respectively). A non-significant difference was marked as n.s. N = technical replicates. RLU: relative luminescence unit. Dashed line: signal-ratio = 1.

Finally, we also aimed to verify the selectivity of the established PCA system. Thus, we stimulated cells which were co-transfected with the favored pair of vectors with different concentrations of 8-Br-cAMP instead of 8-Br-cGMP (Figure 7). Unexpectedly, we observed highly significant signal amplifications following all stimulations comparable to signal amplifications detected after stimulation with 8-Br-cGMP. As depicted on the inset of the figure, the signal-ratio rises highly significant with increasing concentrations.

### 2.8. Dimerization of cGKIα upon Oxidant Stimulation

**Figure 8:**
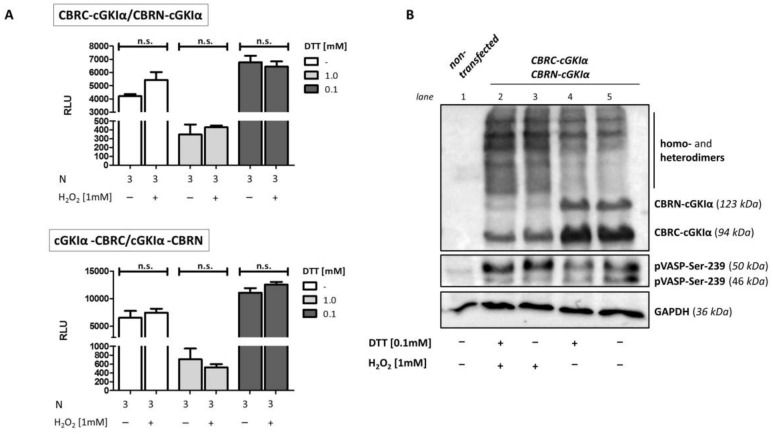
H_2_O_2_-induced cGKIα dimerization. COS7-cells were either seeded in 6-well plates (3.3 × 10^5^ cells/well, (**A**)) or in 75 cm^2^ culture flasks and co-transfected with the vector pairs CBRC-cGKIα/CBRN-cGKIα (**A**,**B**) or cGKIα-CBRC/cGKIα-CBRN (**A**) ((**A**) 1.8 µg DNA, 1:1, B: 22.5 µg, 1:1). 2 h following transfection, dithiothreitol (DTT) (concentration as indicated) was added to medium and cells were either transferred to 96-well plates before incubation (1.0 × 10^4^ cells/well, 24 h, (**A**)) or directly incubated in cell culture flasks before cell harvest for Western Blot analysis (48 h, (**B**)). Before cells were stimulated with H_2_O_2_ (10 min), medium was again changed to DTT-free medium. (**A**) Luciferase assay. No change in luminescence signal could be observed in any condition. Data is expressed as mean ± SEM. A non-significant difference was marked as n.s. N = technical replicates. RLU: relative luminescence unit. (**B**) Western blot. Cells-lysis was performed using a maleimide-containing buffer before separating proteins via 10% SDS-PAGE and subsequent Western blotting. Fusion proteins of cGKIα with CBR-fragments were detected using cGKIα-antibodies, activity of cGKIα was shown using a p-Ser239-VASP antibody, GAPDH served as loading control. No difference in monomer- and dimer band intensity could be identified following reducing compared to non-reducing conditions. Stimulation with hydrogen peroxide caused a shift from monomer to dimer bands in both cases and a slightly increased band intensity of the 50 kDa-band from pVASP-Ser-239, indicating an oxidative activation of cGKIα. VASP: vasodilator-stimulated phosphoprotein.

As already indicated, oxidative dimerization is recently discussed as a cGMP-independent way to activate cGKIα. Already under non-oxidizing conditions, this enzyme forms a homodimer, which is held together by its leucine-zipper domain in its regulatory N-terminal region [37]. Therefore, we examined if H_2_O_2_ can induce this dimerization and subsequent activation in the newly established assay, offering a direct possibility of identifying activated cGKIα. The monomers attach in parallel to each other, and so we combined only vectors, in which the CBRC- and CBRN-fragment are fused either both at the N-terminal or both at the C-terminal position of the kinase. We analyzed several parameters that could influence the dimerization, e.g., number of transfected cells, applied DNA amount, or concentration and duration of hydrogen peroxide stimulation. However, we could not reveal significant signal amplifications probably due to high luminescence intensities already under non-stimulating conditions [34]. We supposed that oxidation of Cys42 already occurs without additional H_2_O_2_ stimulation. Accordingly, we performed the assay using dithiothreitol (DTT) in two different concentrations as a reducing agent (Figure 8A). Following 1 mM DTT, both vector combinations showed a decrease in signal intensity compared to non-reducing conditions. In contrast, lowering the DTT concentration to 0.1 mM led to an increase in RLU to values even above the signal obtained with non-reducing conditions. Again, we did not detect a significant reduction of the luminescence intensities already under non-stimulating conditions compared to H_2_O_2_-treated cells. We also analyzed the impact of hydrogen peroxide stimulation on dimerization and activity of cGKIα using the Western blot (Figure 8B). To protect possible disulfide bonds, we used a modified lysis buffer containing maleimide and Triton X-100 instead of lubrol as detergent. Nonetheless, we could not notice a difference between untreated and DTT-pretreated cells. As expected, in both cases, stimulation with H_2_O_2_ led to a shift from monomeric (CBRN-cGKIα and CBRC-cGKIα) to dimeric forms of cGKIα-fusion proteins. Interestingly, we detected dimeric forms already under non-stimulating conditions, which could not be prevented by DTT. pVASP-Ser 239 was used to reveal cGKIα-activity. We showed a slight band-shift of VASP following H_2_O_2_-stimulation, detected with a specific Ser 239 phospho-antibody, indicating that oxidative activation of cGKIα occurred.

## 3. Discussion

The identification of substrates for cGMP-dependent kinases is a major prerequisite for understanding (patho-)physiological actions of these enzymes in a molecular context and hence, for the development of novel therapeutic strategies to overcome diseases like hypertension and coronary heart diseases. Traditional methods to uncover interaction partners range from co-immunoprecipitation, yeast-2-hybrid, cGMP-agarose affinity purification to phosphor-specific approaches like ^32^P-enzyme assays and the use of special antibodies. However, these techniques mostly do not enable the analysis of the dynamic cGK-signaling in cells and tissues. Therefore, we established a cell-based luciferase-complementation assay using the example of the cGKIα-RGS2 interaction. It has been shown previously that the kinase binds directly to and phosphorylates RGS2 leading to its activation and translocation to the plasma membrane [26,29]. Although we investigated a previously known substrate with our assay, we are the first to our knowledge to provide in vivo evidence of the cGKIα-RGS2-interaction. Initially, we figured out the optimal vector combination by comparison of the different possibilities following 24 h incubation after transfection with or without 8-Br-cGMP stimulation (Figure 3). As we only analyzed cGKIα-RGS2 interaction, we cannot be sure if the position of the CBR tags also holds true for other interactions. Therefore, the vector combination has to be determined all over again when investigating novel interaction partners of cGKIα. We examined the influence of different stimuli in varying concentrations (Figure 5). For this purpose, we analyzed luminescence amplification following stimulation with 8-Br-cGMP vs. 8-pCPT-cGMP using different concentrations. We could not detect a significant difference in signal increase between the two most commonly used concentrations of both analogues (1 mM 8-Br-cGMP vs. 200 µM 8-pCPT-cGMP). In contrast to the bromine-substituted substance, 8-pCPT-cGMP has a more lipophilic structure, enabling it to permeate the cell membrane more easily. Accordingly, a 5-fold lesser concentration led to the same signal intensity. Another reason might be that 8-pCPT-cGMP has the advantage of a much higher resistance to hydrolysis by phosphodiesterases compared to 8-Br-cGMP [38].

Next to different, more general negative controls such as transfection without DNA etc., we also checked the specificity of the system via transfection of two fusion proteins, which are not thought to interact with each other (Figure 6). Unexpectedly, we observed a strong luminescence signal after transfection with CBR-fusion proteins of cGKIα and FK506 binding protein (FKBP), which even slightly intensified (1.3 fold increase) after stimulation with 8-Br-cGMP. However, this increase, although significant, was essentially weaker compared to the signal ratios obtained from the cGKIα/RGS2-constructs (3.6 fold, compare also Figure 3 and Figure 5). We concluded that we noticed an unspecific binding of these two proteins. Wang et al. showed that FKBP-12, a small-size FKBP family member which contains only the FK506-binding domain, binds to a glycine- and serine-rich domain (GS-motif) of transforming growth factor β [39]. In our expression system, we also used GS-motifs to link CBR fragments and target proteins to each other. Hence, the observed interaction between cGKIα-CBRC and CBRN-FKBP may be explained by an unspecific binding of FKBP to the linker-sequence in the cGKIα-CBRC fusion protein, bringing the CBRC- and CBRN-fragments in close proximity and leading to complementation. The conspicuously high signal intensity already under non-stimulating conditions supports this thesis, further evidence for which comes from the fact that cells, which were transfected with cGKIα-CBRN and FRB-CBRC, do not generate an appreciable luminescence signal.

Moreover, we also analyzed the specificity of the system using different concentrations of 8-Br-cAMP (Figure 7). Surprisingly, we discovered a highly significant signal increase in all tested conditions comparable or even slightly higher than the values obtained upon 8-Br-cGMP stimulation. Discrimination between the two second messengers cAMP and cGMP is a critical feature of PKA and PKG. Nevertheless, it has been shown in numerous publications starting in the early 1980s that cGKs can be cross-activated by cAMP, at least in vascular smooth muscle cells [40,41,42,43]. It has been postulated that the selectivity of PKG and PKA is due to only one single amino acid exchange (A/T) in the cyclic nucleotide-binding domain [44]. Moreover, the N-terminal cyclic nucleotide-binding domain (CNBD-A) of human cGKI binds both cGMP and cAMP with a relatively high affinity, showing only a two-fold preference for cGMP [45]. In contrast, the C-terminal low-affinity cyclic nucleotide-binding domain (CNBD-B) is highly selective for cGMP binding with an EC_50_ of 215 nM compared to 52 µM for cAMP [46]. Physiological concentrations of cGMP and cAMP are in the range from 0.1–10 µM and about 1 µM, respectively [47,48]. However, the intracellular 8-Br-cAMP-concentration generated via exogenous stimulation is not clear. Here, we used the commonly applicated 8-Br-cAMP concentrations of 0.125 to 1 mM, which is 2- to 20-fold above the postulated EC_50_. Thereby, we cannot exclude the possibility of cross-activation. Another option which has to be taken into account is cross-talk between cAMP and cGMP via their degrading enzymes called phosphodiesterases (PDEs). While some PDE-families are known to be either cAMP- or cGMP-specific, some are also dual-substrate specific, consequently hydrolyzing both second messengers like PDE-1, -2, -3, -10 and -11. By activation or inhibition of such a PDE, cAMP and cGMP can influence the concentration of each other. One interesting example is PDE10A, whose high affinity for cAMP inhibits cGMP hydrolysis very potently so that only a low cAMP-concentration is needed to antagonize intracellular cGMP degradation (IC_50_ = 0.39 µM) [49]. When studying PDE10A-transfected COS-7 cells, the authors also detected endogenous PDE activity. Therefore, an increase in cGMP concentration upon PDE stimulation by high amounts of cAMP might also be responsible for the signal amplifications seen in our luciferase assay. Hence, due to its structural similarity, cAMP used in higher concentrations could also serve as a stimulator for enzyme-complementation assays analyzing the interaction between cGKIα and other proteins. For the proof of specificity, other intracellular messengers should be used in future.

Finally, we turned towards an alternative stimulation of cGKIα distinct from cyclic nucleotides. In recent years, the point of view that H_2_O_2_ is an unwanted, toxic by-product of aerobic modes of life has changed to a more beneficial function. Meanwhile, this substance is thought to work as part of so-called “redox-signaling” in mammalian cells, whereby different physiological responses such as cell proliferation, differentiation and migration can be mediated (summarized in [50]). cGKIα (in contrast to the β-isoform) can also serve as a redox sensor in cells, since the kinase dimerizes upon oxidative stress by forming an intermonomeric disulfide bond at Cys-42. During in vitro kinase assays using Glasstide as an artificial substrate, hydrogen oxide led to a reduced K_a_ of cGKIα (from 247 to 36 µM) without influencing Vmax [22]. Using a “redox-dead” cGKIα-knock-in mouse as a model, more information is collected concerning the in vivo consequences and, thus, physiological role of this new activation pathway. The workgroup around Philip Eaton showed inter alia an involvement in blood pressure regulation and in cardiac diastolic relaxation, thereby fine-tuning the Frank–Starling response [51,52]. In our lab, we also observed a highly significant increase of cGKIα dimerization upon stimulation with H_2_O_2_ in primary mesangial cells under non-reducing conditions: While only 30% of the kinase is present as a dimer under control conditions, H_2_O_2_ massively enhances the dimerization up to 90% (Figure S3). Accordingly, we wanted to figure out whether we can use the herein established assay to recognize dimerization of cGKIα upon oxidant stimulation (Figure 8A). Although we tried different conditions like variation of time and concentration for stimulation, we could not detect any signal amplification when we co-transfected both CBRC- and CBRN-containing cGKIα-vectors. Instead, we observed very high luminescence signals already under non-stimulating conditions. One possible explanation is that the native, non-oxidized enzyme can already dimerize by a non-covalent interaction of the leucine-zipper domains [37]. In this context, it is important to notice that we already naturally lose 50% of the signal due to the design of the system. No complementation can occur if cGKIα-monomers which carry the same CBR-fragment (2× CBRC-cGKIα or 2× CBRN-cGKIα) dimerize. Moreover, while cultivating cells, reactive oxygen species (ROS) can be generated within the context of a “cell-culture shock” (resulting from stresses that cells experience when they are explanted from their natural environment into culture [53]) due to more O_2_ availability and missing antioxidants in culture medium. For instance, while most eukaryotic cells in vivo are exposed to a low O_2_ concentration with oxygen partial pressures ranging from 1 to 10 mmHg, cell culture is performed in a hyperoxide atmosphere in cell incubators (up to 150 mmHg O_2_), thereby multiplying ROS generation. Besides, the culture medium can even be pro-oxidant for itself, as it contains diverse components like metal ions, which serve as powerful catalysators for forming free radicals (the problem of oxidative stress in cell culture is comprehensively reviewed in [54]). Hence, we tried to prevent this oxidative stress-induced dimerization of cGKIα already under non-stimulating conditions by adding DTT to the cell-culture medium. However, when using 1 mM DTT we observed massively reduced luminescence signals compared to non-DTT-treated cells, which could not even be amplified by the addition of H_2_O_2_ as a stimulus. Hua Long et al. showed that thiol compounds can directly interact with different cell-culture media (also Dulbecco’s modified Eagle medium (DMEM)) and that their oxidation generates hydrogen peroxide in considerable amounts [55]. As enhanced ROS production induces DNA damage and, subsequently, cytotoxic effects, we hypothesized that addition of 1 mM DTT might have led to cytotoxicity and thereby decrease of luminescence. However, when using a 10-fold lower DTT-concentration, we detected even higher luminescence signals compared to non-DTT-treated cells (underlining a function for H_2_O_2_ in cell proliferation [56]), but with 0.1 mM DTT, we could not prevent the basal stimulation at all. It should be mentioned that the luciferase-detection reagent used also contains DTT in an amount not further defined by the manufacturer. Accordingly, effects produced by H_2_O_2_ stimulation were possibly destroyed prior to measurement of luciferase activity by resetting the kinase in a reduced state after addition of this reagent. We also checked cGKIα dimerization and activation in a Western blot (Figure 8B). Here, we did not find any differences between cells pre-treated with 0.1 mM DTT compared to non-treated. Again, in contrast to the results produced with the luciferase assay, following treatment with H_2_O_2_, the band intensity of monomers decreased with a concomitant increase of dimer bands. Moreover, slightly enhanced VASP phosphorylation following hydrogen peroxide incubation suggested at least a weak activation of cGKIα. Notably, we revealed distinct dimer bands even under non-stimulating conditions, a reproducible phenomenon in our laboratory but seen previously by others, too [22,57]. This Western blot analysis also reflected results produced with the luciferase assay, as luminescence signals were appropriately high. Therefore, we assume that the PCA system was already saturated by the background signal produced by the basal interaction, so that stimulation with H_2_O_2_ could not enhance the signal further. However, in a recent work by Kalyanaraman et al. the authors did not find any relation between oxidation-induced disulfide formation and activation of cGKIα at all. In contrast, they stated that a loss of the redox-sensitive cysteine only leads to a reduction in cGMP affinity [25]. Others report on redox-dependent changes in intracellular location of the kinase: It has been shown that H_2_O_2_-treatment of myocytes leads to a translocation of cGKIα to the plasma membrane. While WT-cGKIα redistributed to the cytosol after some time, the so-called “redox-dead” C42S-cGKIα mutant remained predominantly at the membrane [58]. Therefore, a lot more work has to be done to define the exact function and physiological importance of the non-canonical activation pathway of cGKIα. For the aforementioned reasons, our established system is not suitable for the evaluation of enhanced dimerization of cGKIα by hydrogen peroxide. Nonetheless, the CBRN-/CBRC complementation coupled with cGKIα and RGS2 revealed stable interaction of these proteins that is enhanced by cGMP and cAMP. Hence, the PCA can be potentially used to gain a deeper insight into this protein interaction, e.g., with regard to temporal and spatial changes by performing real-time assays [31]. Another aspect could be to mutate the phosphorylation sites of RGS2 and check if the phosphorylation by cGKIα at these sites somehow influences the interaction of both proteins. Moreover, the system could serve as a valuable tool to identify novel protein interactions and, hence, phosphorylation targets of this kinase.

## 4. Materials and Methods

### 4.1. Materials

Materials required for cloning of vectors: Matrices for PCR: pMT3_cGKIα containing cDNA of cGKIα [59], pCMV6_RGS2, containing cDNA of RGS2 (OriGene Technologie, Rockville, MD, USA). Phusion^®^ HF DNA polymerase as well as all restriction enzymes, T4 DNA ligase and used buffers: New England BioLabs (Frankfurt am Main, Germany). dNTPs, DNA loading dyes and size markers: Thermo Scientific (Karlsruhe, Germany). QIAquick Gel extraction kit and plasmid preparation kits: Qiagen (Hilden, Germany). Primers were synthesized by Eurofins Genomics (Ebersberg, Germany), a detailed table regarding used primer sequences can be found in the supplementary material. Used bacteria for amplification: *E. coli* TOP10 (Invitrogen/Thermo Fisher Scientific, Rockford, IL, USA). Full length CBR vectors were kindly provided by T. Ozawa, CBRN and CBRC split vectors were generated by Timo Littmann [30].

Materials required for cell culture and transfection: cell-culture medium: DMEM—high glucose (D6546) and DMEM—Base (D5030) as well as PBS (Sigma-Aldrich Chemie, Steinheim, Germany). Serum: FBS superior, Biochrom GmbH (Berlin, Germany). The following supplements were all obtained from Sigma-Aldrich (Taufkirchen, Germany): antibiotics: 100 U/mL Penicillin G, 100 µg/mL Streptomycin (P4333); MEM non-essential amino acid solution (100×), l-Glutamine solution (200 mM). Trypsin: Trypsin-EDTA-solution (10×) (Sigma-Aldrich, Taufkirchen, Germany).

Materials required for Western blotting: Lowry protein concentration determination kit: Dc protein assay (Bio-Rad Laboratories GmbH, Munich, Germany). PVDF-Membrane: Immobilon (Millipore GmbH, Schwalbach, Germany. Antibodies: cGKIα: 1:500, rabbit, own production [60]; RGS2: 1:500, mouse (Santa Cruz Biotechnology, Heidelberg, Germany); p-VASP Ser239: 1:1000, rabbit (Cell Signaling Technology, Cambridge, UK), β-Actin: 1:2500, rabbit (Abcam, Cambridge, UK); vinculin: 1:500, mouse (R&D Systems, Wiesbaden-Nordenstadt, Germany); GAPDH: 1:1000, rabbit (Cell Signaling Technology, Cambridge, UK); secondary antibodies: mouse IgG HRP-conjugated (1:10000, Dianova GmbH, Hamburg, Germany); rabbit IgG HRP-conjugated (1:25,000, Dianova GmbH, Hamburg, Germany).

Materials required for Luminescence assays: 96-well plates: CELLSTAR^®^ 392-0024 (VWR International. Darmstadt, Germany). Educt for luciferase reaction: Bright-Glo^TM^ luciferase assay system (Promega, Mannheim, Germany). Rapamycin: kind gift from A. Buschauer (Department of Pharmaceutical/Medicinal Chemistry, University of Regensburg). 8-Br-cAMP: Sigma-Aldrich (Taufkirchen, Germany). 8-Br-cGMP and 8-pCPT-cGMP: Biolog Life Science Institute (Bremen, Germany).

### 4.2. Construction of Vectors

The amplification of required DNA-sequences of cGKIα- and RGS2-cDNA was performed via PCR using the aforementioned vectors as matrices and different combinations of primers (as indicated in Table S1) along with simultaneous attachment of different restriction sites for subsequent cloning. Phusion HF DNA polymerase was used to avoid amplification of false nucleotides. Amplificates were purified using a GelExtraction Kit and cloned into pcDNA-vectors containing *click beetle* luciferase fragments using standard methods. All generated vectors were sequenced by Eurofins Genomics (Ebersberg, Germany) to verify correct sequences.

### 4.3. Cell Culture and Transfection

For this study, COS-7 cells were cultured in DMEM supplemented with 10% FCS and aforementioned antibiotics and supplements at 37 °C in a 5% CO_2_ incubator to a confluency around 50%. Cells were then serum starved for 2 h and transfected with different luciferase or control vectors using calcium phosphate transfection according to standard methods [61]. For Western blotting, cells were transfected in 75 cm^2^ flasks, whereas serum was added again 16 h post transfection; 32 h later, cells were harvested (see below). For luminescence assays, cells were transfected in 6-well-plates and incubated for 16–18 h. Afterwards, cells were trypsinated, washed, transferred onto a 96-well-plate (1–2 × 10^4^ cells/well, overall volume 100 µL) and cultured until cell adhesion occurred (approximately 5 h). Cells were then exposed to different stimuli, so that the luminescence assay could be carried out.

### 4.4. Western Blotting

To control expression of transfected vectors, cell harvest was performed using cell scrapers and detergent containing buffer (2% Lubrol, 20 mM Tris, 150 mM NaCl, pH 8.0; containing protease inhibitors Leupeptin (0.5 µg/mL), Benzamindine (1 mM) and PMSF (0.3 mM)). For the analysis of H_2_O_2_-induced dimerization of cGKIα, cells were lysed with a maleimide-containing buffer (25 mM HEPES, 100 mM NaCl, 1 mM EDTA, 100 mM maleimide, 10% (*v*/*v*) glycerol, 1% (*v*/*v*) Triton X 100). Cells were homogenized with a 30-gauge needle and centrifuged (16500× *g*, 4 °C, 10 min). For the comparison of expression in transfected cells and tissue, the cerebellum and colon of a male, 10-week-old 129/Sv wild-type mouse were removed. Organs were washed with cold phosphate buffered saline and homogenized by ultrasonification in Lubrol-lysis buffer (receipt as described above) followed by centrifugation (15000× *g*, 4 °C, 10 min).

Protein concentration of cell- and tissue lysates was determined using an adapted version of the method of Lowry [62]. After denaturing, 30–55 µg of protein/lane was separated in 11.5–12.5% SDS-PAGE and subjected to Western blotting using the aforementioned dilutions of different antibodies. The horse radish peroxidase (coupled to secondary antibodies) activity was visualized using Clarity™ Western ECL Blotting substrate (Bio-Rad Laboratories GmbH, Munich, Germany) with a chemiluminescence detector and its appropriate software (ChemiDoc MP System with ImageLab, Bio-Rad Laboratories GmbH, Munich, Germany).

### 4.5. Luminescence Assay

Following incubation of cells in 96-well-plates, 70 µl of medium/well were replaced by DMEM + FCS but w/o phenol red and cells were cultured for another 24–48 h. Medium contained different stimuli at different concentrations: 8-Br-cGMP: 0.5 mM, 1 mM, 2.5mM; 8-pCPT-cGMP: 100 µM, 200 µM, 500 µM; 8-Br-cAMP: 0.125 mM, 0.25 mM, 0.5 mM, 1.0 mM). Subsequently, 50 µL medium/well were replaced with Bright-Glo™ Luciferase reagent. Plates were then measured in a luminescence capable plate reader (GENios Pro, Tecan Trading AG, Maennedorf, Switzerland) after 2 min of shaking for a 1000 ms measurement time/well. The emitted photons per second were detected (stated as “Relative Luminescence Unit RLU”) and normalized using the XFLuor4GeniosPro software, version 2 (Tecan Trading AG, Maennedorf, Switzerland).

### 4.6. Statistical Analysis

Results are expressed as mean ± SEM, whereas *n* represents technical replicates. Calculation of statistical differences was performed using GraphPad Prism 5. Unpaired Student’s *t*-test (two-tailed, confidence interval 95%) was used for calculation of significant differences between two groups, characterized in figures by *(with Welch’s correction if unequal variances were assumed, then characterized in figures by ^+^). *p*-values < 0.05 were considered significant (*, ^+^), <0.01 and <0.001 highly significant (**, ^++^ and ***, ^+++^, respectively), a non-significant difference was marked as n.s. For calculation of significant differences between more than 2 groups, one-way analysis of variance (ANOVA) followed by the Bonferroni post-test was performed with the same levels of significance as above (characterized in figures by ^+^, ^++^ and ^+++^).

## Supplementary Materials

Supplementary materials can be found at http://www.mdpi.com/1422-0067/19/4/1180/s1.
