# Supplementary material for: Establishing a Split Luciferase Assay for Proteinkinase G (PKG) Interaction Studies
Source: Int J Mol Sci. 2018 Apr 12;19(4):1180. doi: 10.3390/ijms19041180 (PMC5979328; doi:10.3390/ijms19041180)
Supplement: Supplementary file 1 [file ijms-19-01180-s001.pdf]

Supplementary Materials

Table S1. Primers for cloning. Inserted restriction sites are highlighted in bold. Amplification of DNA for cloning of cGKIα-vectors was performed as tandem-PCRS whereas the second PCR was run with the forward-primer containing the restriction site (see also in schematic illustration below table). If necessary, DNA-sequences were purified following agarose-gel-electrophoresis and subsequent methylene-blue-staining using QIAquick Gel extraction kit (Qiagen, Hilden, Germany).

Name	Sequence [5'-->3'], Restriction Site	Target cDNA	Amplified Sequence Used for Cloning of Vector:
MT1_1_f	5'-AGTGGCATGAGCGAGCTG-3'	cGKIα	CBRC-L-cGKIα and CBRN-L-cGKIα
MT1_2_f	5'-GGCGGATCCAGTGGCATG-3' : BamHI	cGKIα	CBRC-L-cGKIα and CBRN-L-cGKIα
MT2_1_r	5'-GAACCGCGGGCCTTAGAAGTCTAT-3' : SacII	cGKIα	CBRC-L-cGKIα and CBRN-L-cGKIα
MT3_1_f	5'-GCCGCCATGAGCGAG - 3'	cGKIα	cGKIα-L-CBRC and cGKIα-L-CBRN
MT3_2_f	5'-TATGCTAGCGCCGCCATG-3' : NheI	cGKIα	cGKIα-L-CBRC and cGKIα-L-CBRN
MT4_1_r	5'-ACTCGAGCCGAAGTCTATGTC - 3' : XhoI	cGKIα	cGKIα-L-CBRC and cGKIα-L-CBRN
MT4_2_r	5'-ACCACCACTCGAGCCGAA-3' : XhoI	cGKIα	cGKIα-L-CBRC and cGKIα-L-CBRN
MT10_f	5'-CGACTGGATCCGTACCGAGG-3' : BamHI	RGS2	CBRC-L-RGS2 and CBRN-L-RGS2
MT6_r	5'-GAACCGCGGTCATGTAGCATGAGG-3' : SacII	RGS2	CBRC-L-RGS2 and CBRN-L-RGS2
MT7_f	5'-TTTAAACTTAAGCTGCCGCCGCGATCG-3' : AflII	RGS2	RGS2-L-CBRC and RGS2-L-CBRN
MT9_r	5'-TTAAAACTCGAGGTTGTAGCATGAGGCTC-3' : XhoI	RGS2	RGS2-L-CBRC and RGS2-L-CBRN

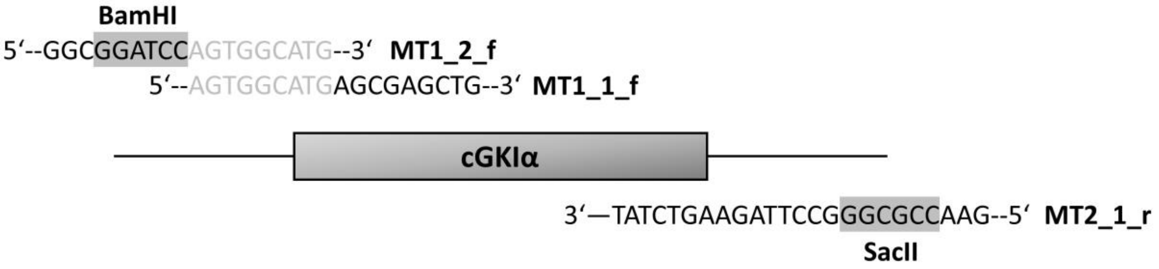

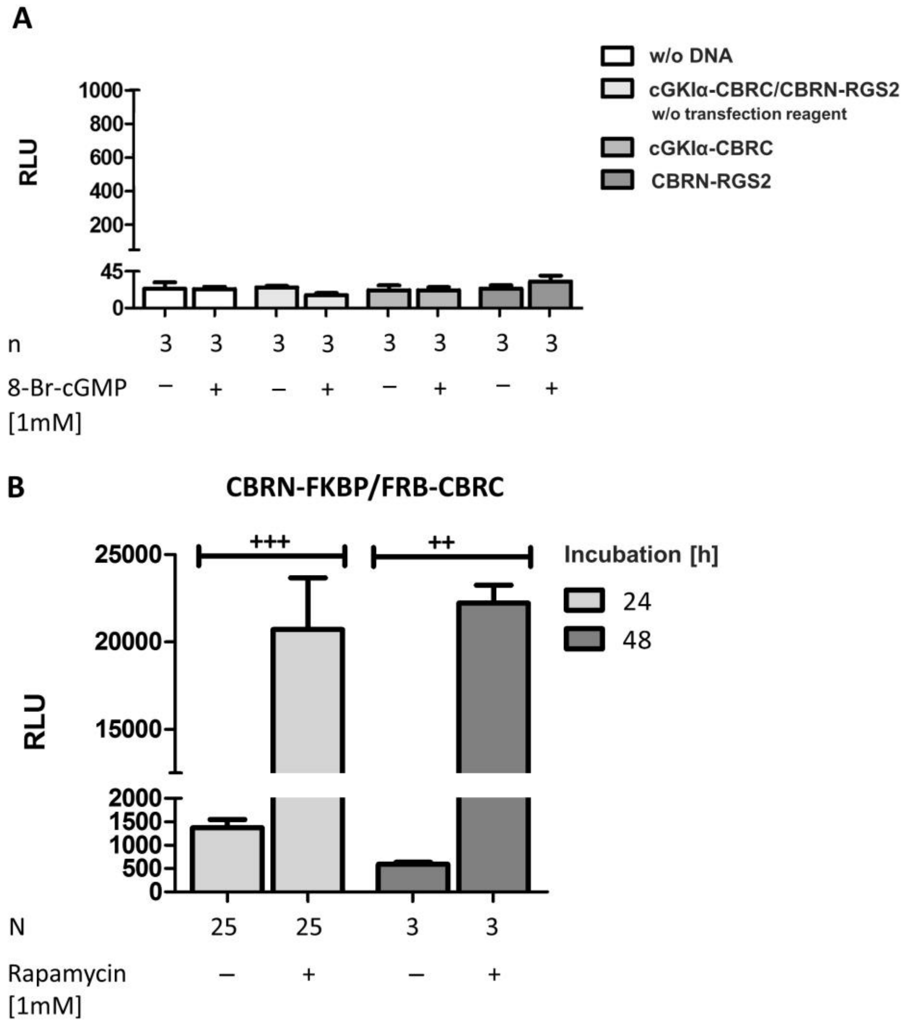

Figure S1. Establishment of controls for luciferase assay. A: Negative controls. COS7-cells were seeded in 6-well plates (3.3 * 10^5 cells/well) and transfected with different controls (150 ng each). After transfer on 96-well plates (1.0 * 10^4 cells/well) and addition of 8-Br-cGMP, cells were incubated for 24 h. **B: Positive controls.** COS7-cells were seeded in 6-well plates (3.3 * 10^5 cells/well) and transfected with 4.5 µg DNA, vector ratio 1:1. After transfer on 96-well plates (1.0 * 10^4 cells/well) and addition of rapamycin, cells were either incubated for 24 or 48 h. A highly significant signal-increase could be observed in both conditions. Data is expressed as mean ± SEM. For unpaired Student's *t*-test with Welch's correction, *p*-values < 0.01 and < 0.001 were considered highly significant ($^{++}$ and $^{+++}$, respectively). N = technical replicates. RLU: relative luminescence unit.

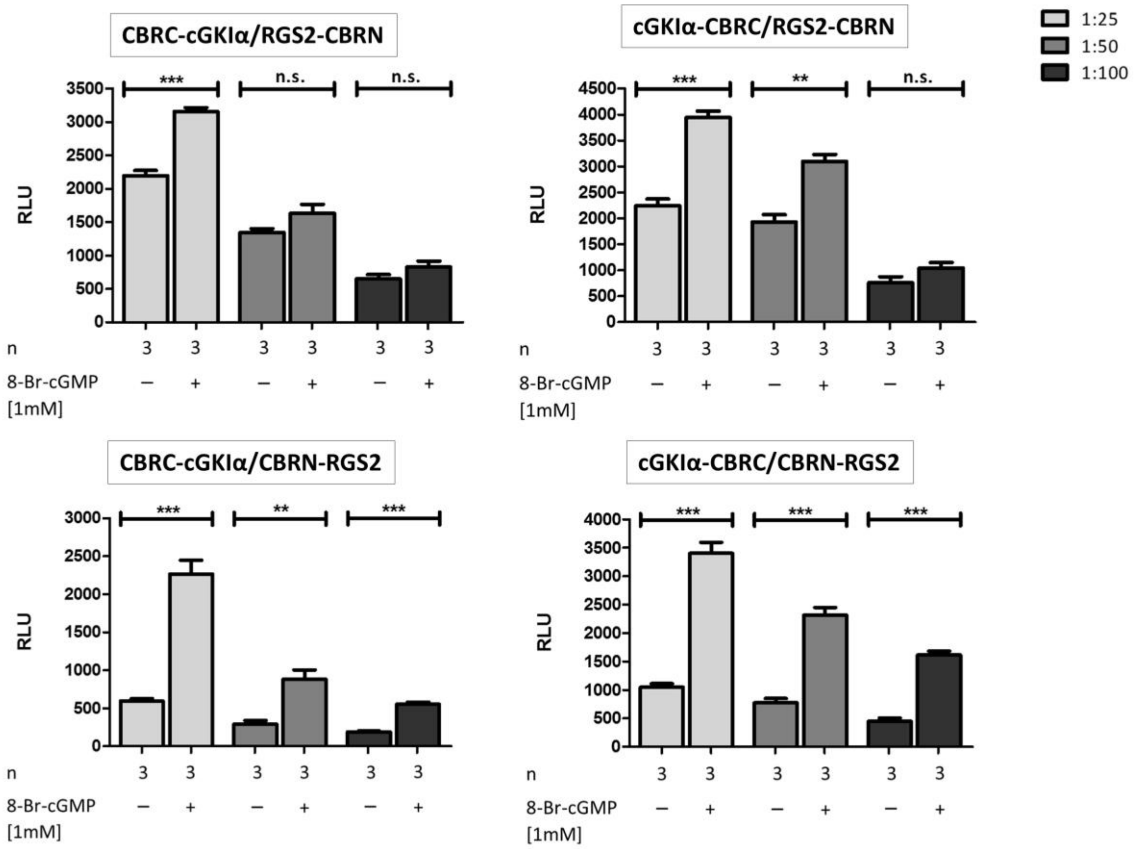

Figure S2. Influence of vector ratio for transfection and subsequent interaction analysis of cGKIα and RGS2 following 48 h incubation. COS7-cells were seeded in 6-well plates ($3.3 * 10^5$ cells/well) and transfected with 4 different combinations of cGKIα/RGS2-vectors (each transfection with 15 µg DNA, vector ratio as indicated). After transfer on 96-well plates ($1.0 * 10^4$ cells/well) and addition of 8-Br-cGMP, cells were incubated for 48 h. In most cases, a significant signal-increase can be observed. Data is expressed as mean ± SEM. For unpaired Student's t-test, *p*-values < 0.01 and < 0.001 were considered highly significant (** and ***, respectively), a non-significant difference was marked as n.s.. N = technical replicates. RLU: relative luminescence unit.

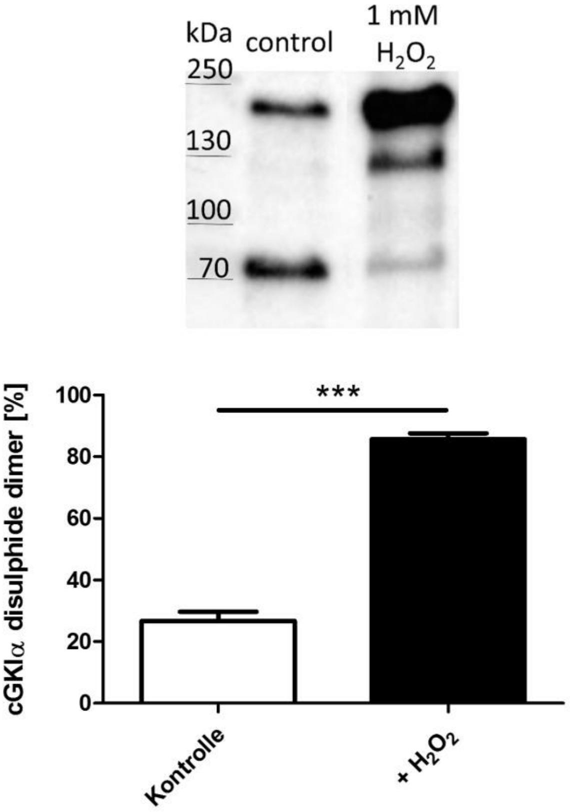

Figure S3. Influence of H_2O_2-treatment on dimerization of cGKI*α*. Primary mesangial cells were isolated and cultured as previously described (Am J Pathol, 2002, 161:799-805). 4 x 10^5 cells were grown in 6-well plates until reaching 90% confluency and either left untreated or stimulated with 1 mM H_2O_2 for 10 minutes. Cell-harvest was performed in 80 µl of a non-reducing, maleimide-containing lysis buffer and 20 µl of protein lysates were analyzed using SDS-PAGE and Western blot using cGKIα-antibodies. Compared to control cells, a highly significant increase in dimerization upon H_2O_2-treatment can be observed. Data is expressed as mean ± SEM. For unpaired Student's t-test *p*-values < 0.001 were considered highly significant (***). N = 7 each, biological replicates.
